# Factors associated with consuming unhealthy food in school children: A population‐based study from Hong Kong

**DOI:** 10.1002/hsr2.1964

**Published:** 2024-03-11

**Authors:** Junjie Huang, Sze Chai Chan, Wing Sze Pang, Shui Hang Chow, Yat Ching Fung, Vera M. W. Keung, Calvin K. M. Cheung, Amelia S. C. Lo, Lancelot W. H. Mui, Albert Lee, Martin C. S. Wong

**Affiliations:** ^1^ Centre for Health Education and Health Promotion, Faculty of Medicine The Chinese University of Hong Kong Hong Kong SAR China; ^2^ Jockey Club School of Public Health and Primary Care, Faculty of Medicine The Chinese University of Hong Kong Hong Kong SAR China; ^3^ The School of Public Health Peking University Beijing China; ^4^ The School of Public Health The Chinese Academy of Medical Sciences and The Peking Union Medical Colleges Beijing China; ^5^ The School of Public Health Fudan University Shanghai China

**Keywords:** adolescents, overweight, students, unhealthy diet, unhealthy food

## Abstract

**Background and Aims:**

Unhealthy diets were found to be the main contributor to the overweight problem among adolescents. In this study, we aim to identify the factors causing unhealthy eating habits in adolescents.

**Methods:**

School‐aged children and adolescents participated in this cross‐sectional observational study with additional school and parental consent. A self‐administered survey was conducted by 30 primary schools and 25 secondary schools. Participants were asked about the frequency of consuming unhealthy food and the types of unhealthy food consumed. A descriptive analysis was performed to demonstrate the proportions of characteristics. The prevalence of the outcome among participants of various factors was also analyzed using separate binary regression models.

**Results:**

A total of 4884 responses were collected. Among primary school students (grade 4, mean age: 10.06), people who (1) were actively gaining weight (aOR: 1.651, 95% CI 1.006–2.708, *p* = 0.047), (2) went to bed after 11 p.m. (aOR: 1.652, 95% CI 1.065–2.563, *p* = 0.025), (3) had more than 2 h of gaming (aOR: 2.833, 95% CI 1.913–4.195, *p* < 0.001), (4) suffered from self‐report depressive symptoms (aOR: 1.753, 95% CI 1.233–2.493, *p* = 0.002) was more likely to consume unhealthy food. As for secondary school students (grade 3, mean age: 15.28), (1) males (aOR: 1.266, 95% CI 1.0004–1.601, *p* = 0.0496), (2) average‐to‐high socioeconomic status (Average: aOR: 1.471, 95% CI 1.115–1.941, *p* = 0.006; High: aOR: 2.253, 95% CI 1.585–3.202. *p* < 0.001), (3) having more than 2 h of gaming (aOR: 1.342, 95% CI 1.069–1.685, *p* = 0.011), (4) suffering from psychological distress (aOR: 1.395, 95% CI 1.051–1.852, *p* = 0.021) were associated with the increased odds of consuming unhealthy food.

**Conclusion:**

Several lifestyle and health factors were significantly associated with unhealthy eating behaviors in school‐aged children and adolescents in Hong Kong, sharing similarities with many other countries. In conjunction with implementing a policy that addresses factors for unhealthy eating habits, further research should investigate potential interventions targeting these factors to ultimately tackle the overweight and obesity concern for children and adolescents in Hong Kong.

## Highlights

- Unhealthy eating habits continue to be an important public health concern among young students in Hong Kong, a fifth of whom were recently reported as overweight or obese in Hong Kong.
- The current study has identified factors and quantitatively measured the strengths of their associations with unhealthy eating behaviors in school‐aged children and adolescents in Hong Kong.
- The factors identified in this study can help relevant authorities make targeted interventions or policy decisions to solve the problem of overweight or obesity among school students.

## 1 INTRODUCTION

Unhealthy food consumption has been a main public health concern among school‐aged children which contributes to obesity and other chronic diseases.^1^ Unhealthy foods include fried foods, canned foods, processed meats, and sugar‐sweetened beverages which are high in energy, high in salt, and high in sugar.^2^, ^3^, ^4^ In Hong Kong, children have been found to have unbalanced diets, including excessive meat and grain consumption.^5^ Adolescents in Hong Kong often consumed unhealthy foods, lacked sufficient fruits and vegetables, and did not find healthy eating enjoyable.^1^, ^6^, ^7^, ^8^ Another qualitative study reported that adolescents did not consider health as the top priority when making food choices; those with fewer healthy eating habits had a poor perception of health risk.^7^ Unhealthy eating habits in adolescents were also affected by peer influence, lack of knowledge of and easy access to healthy food, and the perception that ‘healthful food is more expensive’.^1^, ^7^

Although the Department of Health in Hong Kong^9^ has recommended food intake for school children is in the ratio of 3:2:1 (grains:vegetables:meat and its alternatives) by volume, the evident proportion of school‐aged children with overweight or obesity reflects the serious problem of unhealthy food consumption young students.^10^ The latest local data showed that, in the annual school year of 2019/2020, the percentage of overweight and obesity of primary school and secondary school students is 19.0% and 21.3%, respectively.^11^ The evidence supports the necessity to discover the potential associated factors of unhealthy food consumption in local young age students.

For young students, there are multiple factors associated with unhealthy food intake. Unsecure food environment is a challenge to maintain healthy eating habits.^12^ For example, fast food restaurants are usually located near schools, its convenience and cheaper food price will let more children eat more unhealthy foods.^13^ Regarding activities, TV viewing and gaming screening time have been associated with a higher likelihood of unhealthy food consumption.^14^ The tendency to choose high‐calorie food when watching TV and playing computer games, the longer screen time the school‐aged children spend, the effect of more unhealthy food consumption occurs.^15^ On the other hand, skipping breakfast is another factor that affects school‐aged children developing an unhealthy dietary pattern.^16^ Also, poor health condition is linked to unhealthy food intake for younger students.^17^, ^18^, ^19^ The overconsumption of junk food would affect young adolescents' mental health, such as anxiety and depression.^18^, ^19^ Besides, especially primary school students, they are easy to be caught attention by the foods packed with bright color and cartoon characters.^20^ It could be possible for children to consume more unhealthy foods, which may be harmful to their body weight when they do not recognize the importance of healthy eating.^21^

Previous studies regarding school children's unhealthy food consumption are frequently discussed in other countries, mostly in Europe, North America, and Oceania,^3^, ^12^, ^13^, ^19^ but are limited in the Hong Kong context.^1^, ^2^ There have been a few small‐scale studies in Hong Kong which primarily focused on children and adolescent interviews regarding potential factors, attitudes, and perceptions towards unhealthy eating habits;^1^, ^5^, ^6^, ^7^, ^8^ however, there has not been a comprehensive quantitative analysis of associated factors conducted on a large cohort of this population in Hong Kong thus far. This study aims to identify the factors associated with unhealthy food intake among school‐aged children in both primary and secondary school, through assessing socio‐demographic factors, health‐related behaviors, and their social relationship. It will help to recognize the vulnerable adolescents with unhealthy eating habits and to implement feasible intervention measures to facilitate healthier food consumption.

## 2 METHODS

### 2.1 Participant recruitment

In this study, students were recruited from local primary and secondary schools which participated in the project “Quality Education Fund Thematic Network on Health Schools.” The project conducts school health assessments, seminars and surveys for participating schools to identify the health risks of students to support and promote targeted improvements in schools. The project was carried out by the Centre for Health Education and Health Promotion of the Chinese University of Hong Kong (CHEHP) with 30 primary schools and 25 secondary schools participating in the survey.

### 2.2 Data collection

The procedure of survey collection was divided into several steps. First and foremost, students were informed that their participation was voluntary and the collection of data would be conducted in an anonymous way to safeguard the privacy of students. The confidentiality of students' answers was guaranteed to students and their grades would not be affected by their participation or responses given. Secondly, parental consent and school consent were both collected before conducting the survey. Thirdly, the interviewers who were responsible for the purpose and feature explaining to the students were given appropriate training. The last step of the data collection was to emphasize the confidentiality of the collected data. At the beginning of the survey, students who participated with parental consent were identified with the help of a class teacher, and were not allowed to answer queries from students and access the questionnaire responses.

### 2.3 Survey instruments

An expert panel, including epidemiologists, healthcare professionals, and physicians, were participated and approved the validation of the survey by using the pilot tests. The questionnaire was pilot‐tested in one primary school and two secondary schools on approximately 120 primary and secondary school students for feedback and comments on the clarity, level of difficulty, ease of understanding, wording, and structure. The survey consists of 18 questions in both Traditional Chinese and English versions. Participants' sociodemographic data, including sex, age, number of household computers, ownership of cars (household), ownership of private bedroom, travelling history in the past 12 months, self‐perceived academic performance, self‐reported expectations from parents, and computer ownership (household) (Questions 1 to 9) were collected. Moreover, health‐related parameters were also examined, including self‐perceived health, physical activity, sleeping time, weight control, time spent on TV, games, and social media, psychological distress (using the Kessler Psychological Distress Scale [K6}), and depression (Questions 10 to 15). As for the social relationships, the life satisfaction level of family and friends was used to examine the social relationships of the participants (Question 16). Unhealthy eating habits were measured in terms of consumption frequency of several types of junk food in the last 7 days (Question 17). Lastly, breakfast habits were also examined (Questions 18). The detailed questionnaire has been attached as Supporting Information file 1.

### 2.4 Variables

In the current study, unhealthy foods include (1) crisps or other snacks, (2) chocolate or candies, (3) desserts, ice cream, cake, tart, (4) soft drinks, (5) carton‐packed juice, lemon tea or other sugary drinks, (6) fried food, and (7) processed or preserved meat which are based on nutritional guidelines from the Hong Kong Department of Health.^22^ Respondents were required to answer the consumption frequency of the above seven types of unhealthy food items. Each item provides four options: (1) No, (2) 1–3 times in 7 days, (3) 4–6 times in 7 days, and (4) once or more a day. The consumption of unhealthy food is defined as at least having three of the above unhealthy foods consumed at least four times a week.

Physical health was measured across several domains. Respondents were considered physically active if they completed at least 60 min of moderate or vigorous exercise for 3 days or more in the last 7 days, as per the guidelines from the Centers for Disease Control and Prevention.^23^ Sleeping late has been associated with fewer healthy eating habits in children^24^ and a local study previously showed that the average bedtime for elementary students was around 10 p.m.^25^ suggesting a reasonable cut‐off time of 11 p.m. for late sleepers in the current study.

The Family Affluence Scale (FAS) was used to measure socioeconomic status. Due to insufficient knowledge and unwillingness to disclose relevant information about children and adolescents, gathering precise information about their socioeconomic status might be difficult. The question on ownership of private bedrooms was included in this study because it is one of the useful indicators for assessing the socioeconomic status of the students,^26^ and socioeconomic status were associated with snacking among adolescents in many past cross‐sectional studies.^27^, ^28^ Self‐perceptions of academic performance may cause stress and anxiety among students;^29^ however, anxiety and pressure have been linked to unhealthy eating behaviors.^30^ Therefore, academic performance is an important variable to understand whether students' self‐perception and psychological factors have an impact on their unhealthy eating behaviors. To evaluate the mental health factors of unhealthy food consumption, two negative health outcomes, which were psychological distress and depression, were included. By using the Kessler Psychological Distress Scale (K6), nonspecific psychological distress with six questions on feelings of nervousness, hopelessness, restlessness or fidgety, worthlessness, so depression that nothing could cheer one up and that everything was a burden were evaluated.^31^ Each question contained five options and participants were required to be one of the five options in each sub‐questions: none of the time (0), a little of the time (1), some of the time (2), most of the time (3), and all of the time (4), with points allocated to each option accordingly. The total score is 24, while the cut‐off score is 13 or above.^32^ Detailed coding of the variables has been listed in Supporting Information file 2.

### 2.5 Statistical analysis

To analyze the received data, IBM Statistical Package for Social Sciences (SPSS) software version 26.0 was used. By using the software, the descriptive analysis was conducted to examine the characteristics of the study subjects including the disparity between the primary and secondary school students. The characteristic contrast among participants was demonstrated in proportion to the outcome variables. To evaluate the association between factors and outcome variables (unhealthy food consumption), separate binary regression models were designed and adopted. Moreover, after finishing the adjustments, multiple logistic regression models were utilized to evaluate the explanatory association between factors and the outcome variables. Results with *p* value smaller than 0.05 were considered statistically significant.

## 3 RESULTS

### 3.1 Respondents' characteristics

Responses from 4867 respondents were collected with 2223 (45.7%) from primary school students and 2644 (54.3%) responses from secondary school students (Table 1). Among primary school participants, males were the majority group (*N* = 1146, 51.6%). Conversely, females were the majority of the secondary school participants (*N* = 1334, 50.5%). Students with medium socioeconomic status were the majority in both primary (*N* = 1130, 52.5%) and secondary school group (*N* = 1359, 52.1%), while low (primary: *N* = 397, 18.4%; secondary: *N* = 751, 28.8%) and high (primary: *N* = 627, 18.4%; secondary: *N* = 500, 19.2%) socioeconomic status students were the minorities. As for the ownership of private bedrooms, the majority of students in both the primary (*N* = 1216, 55%) and secondary school (*N* = 1365, 51.9%) groups did not own a private bedroom. Regarding academic performance, primary school students with an average academic performance had the highest percentage (*N* = 925, 42.3%) in primary school group, whereas secondary school students with lower academic performance (*N* = 987, 32.9%) were the majority in the secondary school group, the bedtime of primary school and secondary school student demonstrated an opposite distribution. Most of the primary students slept before 11 p.m. (*N* = 1875, 89.4%), whereas more secondary school students slept at 11 p.m. or later (*N* = 1674, 69.6%). The majority of primary school students reported that they were maintaining weight (*N* = 799, 36.4%), while secondary school students reported that they were not actively controlling their weight (*N* = 944, 35.9%). Higher proportion of primary school students reported that they had physical activity (*N* = 1296, 59.9%), whereas most of the secondary school students reported that they had no physical activity (*N* = 1738, 66.1%). Both primary and secondary school students reported that they spent less than 2 h on gaming (primary: *N* = 1795, 81.2%; secondary: *N* = 1371, 52.2%) and social media (primary: *N* = 2048, 93.3%; secondary: *N* = 1577, 60.0%) during weekdays. However, secondary school students reported that they spent more than 2 h on TV (*N* = 1417, 54.0%), while primary school students did not (*N* = 1497, 68.0%). Majority of students in both groups reported that they did not experience depression (primary: *N* = 1451, 70.6%; secondary: *N* = 1828, 70.0%) and psychological distress (primary: *N* = 1939, 90.0%; secondary: *N* = 2135, 81.4%). Similarly, the consumption of unhealthy food such as crisps and other snacks (primary: *N* = 1936, 87.2%; secondary: *N* = 2348, 88.9%), chocolate or candies (primary: *N* = 1825, 82.4%; secondary: *N* = 2048, 77.6%), desserts, ice‐cream, cake or tart (primary: *N* = 1964, 88.7%; secondary: *N* = 2267, 86.1%), soft drinks (primary: *N* = 1999, 90.8%; secondary: *N* = 2136, 81.0%), carton‐packed juice, lemon tea, or other sugary drinks (primary: *N* = 1749, 79.4%; secondary: *N* = 1644, 62.5%), fried food (primary: *N* = 1988, 90.1%; secondary: N = 2334, 88.5%), and processed or preserved meat (primary: *N* = 1888, 85.7%; secondary: *N* = 2117, 80.3%) in both groups. A large proportion of primary school students reported that they had regular breakfast (*N* = 1722, 82.6%), while secondary students reported that they did not have regular breakfast (*N* = 1362, 54.2%). Moreover, the majority of both groups reported that they were satisfied with their school (primary: *N* = 1618, 73.8%; secondary: *N* = 1786, 67.5%) and family life (primary: *N* = 1750, 79.6%; secondary: *N* = 1678, 63.5%).

**Table 1:** Characteristics of the participants.

	Primary school	Secondary school
Sex		
Male	1146 (51.6%)	1310 (49.5%)
Female	1077 (48.4%)	1334 (50.5%)
Socioeconomic status		
Low	397 (18.4%)	751 (28.8%)
Medium	1130 (52.5%)	1359 (52.1%)
High	627 (29.1%)	500 (19.2%)
Ownership of private bedroom		
No	1216 (55.0%)	1365 (51.9%)
Yes	994 (45.0%)	1265 (48.1%)
Academic Performance		
Poor	431 (19.7%)	987 (37.4%)
Average	925 (42.3%)	868 (32.9%)
Good	831 (38.0%)	787 (29.8%)
Bedtime		
Before 11 p.m.	1875 (89.4%)	730 (30.4%)
11 p.m. or later	223 (10.6%)	1674 (69.6%)
Weight control		
No active control	522 (23.7%)	944 (35.9%)
Reducing weight	542 (24.7%)	891 (33.9%)
Maintaining weight	799 (36.4%)	524 (19.9%)
Gaining weight	335 (15.2%)	271 (10.3%)
Physical inactivity		
No	1296 (59.9%)	892 (33.9%)
Yes	867 (40.1%)	1738 (66.1%)
More than 2 h of … during weekdays		
TV		
No	1497 (68.0%)	1205 (46.0%)
Yes	704 (32.0%)	1417 (54.0%)
Gaming		
No	1795 (81.2%)	1371 (52.2%)
Yes	415 (18.8%)	1256 (47.8%)
Social media		
No	2048 (93.3%)	1577 (60.0%)
Yes	146 (6.7%)	1052 (40.0%)
Depression		
No	1451 (70.6%)	1828 (70.0%)
Yes	604 (29.4%)	783 (30.0%)
Psychological distress		
No	1939 (90.0%)	2135 (81.4%)
Yes	215 (10.0%)	489 (18.6%)
Unhealthy food consumption		
Crisps and other snacks		
No	1936 (87.2%)	2348 (88.9%)
Yes	284 (12.8%)	294 (11.1%)
Chocolate or candies		
No	1825 (82.4%)	2048 (77.6%)
Yes	389 (17.6%)	591 (22.4%)
Desserts, ice‐cream, cake or tart		
No	1964 (88.7%)	2267 (86.1%)
Yes	249 (11.3%)	367 (13.9%)
Soft drinks		
No	1999 (90.8%)	2136 (81.0%)
Yes	203 (9.2%)	500 (19.0%)
Carton‐packed juice, lemon tea, or other sugary drinks		
No	1749 (79.4%)	1644 (62.5%)
Yes	453 (20.6%)	985 (37.5%)
Fried food		
No	1988 (90.1%)	2334 (88.5%)
Yes	219 (9.9%)	302 (11.5%)
Processed or preserved meat		
No	1888 (85.7%)	2117 (80.3%)
Yes	316 (14.3%)	520 (19.7%)
Regular breakfast		
Yes	1722 (82.6%)	1149 (45.8%)
No	363 (17.4%)	1362 (54.2%)
Life satisfaction		
School		
No	575 (26.2%)	860 (32.5%)
Yes	1618 (73.8%)	1786 (67.5%)
Family		
No	449 (20.4%)	965 (36.5%)
Yes	1750 (79.6%)	1678 (63.5%)

### 3.2 Prevalence of unhealthy dietary habits

Among primary school students (Table 2), a higher prevalence of unhealthy food consumption was observed in students who are males (15.3% vs. 10.6%, *p* = 0.001), having a low (19.1%) or average academic performance (12.8% vs. 10.3% for good performance), slept after 11 p.m. (24.2% vs. 11.3%, *p* < 0.001), spent more than 2 h on TV (22.1% vs. 8.5%, *p* < 0.001), gaming (31.2% vs. 8.8%, *p* < 0.001), and social media on weekdays (33.3% vs. 11.5%, *p* < 0.001), depressed (8.3% vs. 9.9%, *p* < 0.001), psychologically distressed (21.3% vs. 12.2%, *p* < 0.001), not having breakfast regularly (21.0% vs. 11.3%, *p* < 0.001), and not satisfied with school life (17.3% vs. 11.3%, *p* < 0.001) and family life (16.6% vs. 12.4%, *p* = 0.014).

**Table 2:** Prevalence of unhealthy food consumption.

	Primary school	*p* Value	Secondary school	*p* Value
Overall	279 (13.0%)		515 (19.4%)	
Sex				
Male	169 (15.3%)	0.001*	284 (22.1%)	0.003*
Female	109 (10.6%)		229 (17.4%)	
Socioeconomic status				
Low	40 (10.3%)	0.145	115 (15.6%)	<0.001*
Medium	139 (12.8%)		265 (19.8%)	
High	88 (14.6%)		127 (25.9%)	
Ownership of private bedroom				
No	143 (12.3%)	0.312	245 (18.3%)	0.046*
Yes	132 (13.8%)		266 (21.4%)	
Academic performance				
Poor	79 (19.1%)	<0.001*	210 (21.7%)	0.147
Average	113 (12.8%)		155 (18.2%)	
Good	83 (10.3%)		149 (19.2%)	
Bedtime				
Before 11 p.m.	204 (11.3%)	<0.001*	112 (15.6%)	0.011*
11 p.m. or later	51 (24.2%)		328 (20.0%)	
Weight control				
No active control	64 (12.8%)	0.460	210 (22.5%)	<0.001*
Reducing weight	59 (11.3%)		141 (16.2%)	
Maintaining weight	103 (13.3%)		84 (16.4%)	
Gaining weight	48 (15.1%)		76 (28.7%)	
Physical inactivity				
No	163 (13.1%)	0.910	195 (22.3%)	0.020*
Yes	107 (12.9%)		316 (18.5%)	
More than 2 h of … during weekdays				
TV				
No	123 (8.5%)	<0.001*	198 (15.9%)	<0.001*
Yes	149 (22.1%)		322 (23.0%)	
Gaming				
No	153 (8.8%)	<0.001*	211 (15.7%)	<0.001*
Yes	123 (31.2%)		298 (24.1%)	
Social media				
No	227 (11.5%)	<0.001*	254 (16.4%)	<0.001*
Yes	46 (33.3%)		253 (24.4%)	
Depression				
No	138 (9.9%)	<0.001*	309 (17.2%)	<0.001*
Yes	106 (18.3%)		199 (25.9%)	
Psychological distress				
No	228 (12.2%)	<0.001*	376 (18.0%)	<0.001*
Yes	44 (21.3%)		134 (27.7%)	
Regular breakfast				
Yes	187 (11.3%)	<0.001*	184 (16.3%)	<0.001*
No	73 (21.0%)		300 (22.4%)	
Life satisfaction				
School				
No	96 (17.3%)	<0.001*	185 (21.9%)	0.061
Yes	176 (11.3%)		329 (18.8%)	
Family				
No	72 (16.6%)	0.014*	207 (21.9%)	0.051
Yes	203 (12.1%)		308 (18.7%)	

*Note*: *p < 0.05.

As for the secondary school students (Table 2), a higher prevalence of unhealthy food consumption was observed among students who are male (22.1% vs. 17.4%, *p* = 0.003), having a high (25.9%), or medium socioeconomic status (19.8% vs. 15.6% for low socioeconomic status, *p* < 0.001), owning a private bedroom (21.4% vs. 18.3%, *p* = 0.046), slept after 11 p.m. or later (20.0% vs. 15.6%, *p* = 0.011), gaining weight (28.7%), did not control weight (22.5%), or maintaining weight (16.4% vs. 16.2% for reducing weight, *p* < 0.001), with physical activity (22.3% vs. 18.5%, *p* = 0.020), spent more than 2 h on TV (23.0% vs. 15.9%, *p* < 0.001), gaming (24.1% vs. 15.7%, *p* < 0.001), and social media on weekdays (24.4% vs. 16.4%, *p* < 0.001), depressed (25.9% vs. 17.2%, *p* < 0.001), psychologically distressed (27.7% vs. 18.0%, *p* < 0.001), and having breakfast regularly (22.4% vs. 16.3%, *p* < 0.001).

### 3.3 Factors associated with unhealthy dietary habits

Among primary school students (Table 3), those who slept at or after 11 p.m. were significantly associated with the increased odds of unhealthy food consumption (aOR: 1.65, 95% CI: 1.065–2.56, *p* = 0.025). Gaining weight was also one of the associated factors of unhealthy food consumption (aOR: 1.65, 95% CI: 1.01–2.708, *p* = 0.047). Moreover, more than 2 h of gaming (aOR: 2.83, 95% CI: 1.91–4.20, *p* <0.001), and TV during weekdays (aOR: 1.94, 95% CI: 1.35–2.79, *p*  <0.001), suffered from depression (aOR: 1.75, 95% CI: 1.23–2.49, *p* = 0.002), and not having breakfast regularly (aOR: 1.46, 95% CI: 1.00–2.14, *p* = 0.048) were also positively associated with the consumption of unhealthy food.

**Table 3:** Factors associated with unhealthy food consumption.

	Primary school			Secondary school		
	aOR	95% CI	*p* Value	aOR	95% CI	*p* Value
Sex						
Female	1 (ref)			1 (ref)		
Male	1.238	0.891–1.721	0.203	**1.266**	**1.0004–1.601**	**0.0496**
FAS score						
Low	1 (ref)			1 (ref)		
Average	1.283	0.811–2.030	0.287	**1.471**	**1.115–1.941**	0.006*
High	1.563	0.937–2.609	0.087	**2.253**	**1.585–3.202**	<0.001*
Ownership of private bedroom (ref: no)						
Yes	1.201	0.852–1.693	0.297	0.940	0.738–1.196	0.614
Perceived academic performance						
Excellent or Good	0.875	0.609–1.258	0.472	1.114	0.845–1.468	0.444
Average	1 (ref)			1 (ref)		
Bad or poor	1.032	0.677‐1.574	0.883	1.051	0.809–1.367	0.709
Bedtime						
Before 11 p.m.	1 (ref)			1 (ref)		
11 p.m. or later	**1.652**	**1.065–2.563**	0.025*	1.138	0.874–1.482	0.336
Physical Inactivity (ref: no)						
Yes	0.922	0.662–1.284	0.630	0.805	0.640–1.013	0.065
Weight control						
No active control	1 (ref)			1 (ref)		
Reducing weight	0.773	0.475–1.258	0.300	**0.571**	**0.436–0.749**	<0.001*
Maintaining weight	1.144	0.742–1.762	0.543	**0.708**	**0.523–0.959**	**0.026**
Gaining weight	**1.651**	**1.006–2.708**	0.047*	1.355	0.965–1.903	0.079
More than 2 h of … during weekdays (ref: no)						
Gaming	**2.833**	**1.913–4.195**	<0.001*	**1.342**	**1.069–1.685**	0.011*
Social media	1.574	0.921–2.689	0.097	**1.436**	**1.143–1.805**	0.002*
TV	**1.943**	**1.351–2.794**	<0.001*	**1.326**	**1.055–1.667**	0.015*
Mental health conditions (ref: no)						
Psychological distress	1.154	0.706–1.885	0.567	**1.395**	**1.051–1.852**	0.021*
Depression	**1.753**	**1.233–2.493**	0.002*	**1.509**	**1.174–1.938**	0.001*
Life satisfaction (ref: not satisfied)						
Family	0.827	0.561–1.220	0.338	0.895	0.700–1.144	0.374
School	0.874	0.609–1.254	0.464	0.973	0.754–1.255	0.834
Regular breakfast (ref: yes)						
No	**1.464**	**1.003–2.138**	0.048*	**1.347**	**1.075‐1.688**	0.010*

*Note*: *p < 0.05.

As for the secondary school group (Table 3), males were associated with unhealthy food consumption (aOR: 1.27, 95% CI: 1.000–1.601, *p* = 0.050). Also, who's with high (aOR: 2.25, 95% CI: 1.56–3.20, *p* < 0.001) and average (aOR: 2.25, 95% CI: 1.56–3.20, *p* = 0.006) FAS score was also associated with the unhealthy food consumption. Comparing with the primary school group, reducing weight (aOR: 0.57, 95% CI: 0.436–0.749, *p* < 0.001) and maintaining weight (aOR: 0.71, 95% CI: 0.96–3.20, *p* = 0.026) were negatively associated with the consumption of unhealthy food among secondary school students. On the other hand, gaming, social media, and TV watching for more than 2 h during weekdays were positively associated with unhealthy food consumption. (Gaming: aOR: 1.34, 95% CI: 1.07–1.69, *p* = 0.011; social media: aOR: 1.43, 95% CI: 1.14–1.81, *p* = 0.002; TV: aOR: 1.33, 95% CI: 1.06–1.94, *p* = 0.015.) Students who had psychological distress (aOR: 1.40, 95% CI: 1.05–1.85, *p* = 0.021) and depression (aOR: 1.51, 95% CI: 1.17–1.94, *p* = 0.001) were also having higher odds in unhealthy food consumption. Similar to the primary school group, not having breakfast regularly (aOR: 1.35, 95% CI: 1.07–1.69, *p* = 0.010) was also positively associated with the odds of consuming unhealthy food.

## 4 DISCUSSION

### 4.1 Major findings summary

Both groups of participants shared the same variables of a higher prevalence of unhealthy food consumption patterns, which included more than 2 h of leisure time during weekdays (gaming and TV), suffering poorer mental health conditions in depression, and skipping breakfast regularly. Besides, compared with primary school students, secondary school students had a higher likelihood of unhealthy food consumption when they have average to high Family Affluence Scale (FAS) score, spend 2 h or above on social media, and suffer from psychological distress. For elementary school students, they are more likely to eat unhealthy food if they have a late bedtime (after 11 p.m.), and when they are actively gaining weight.

### 4.2 Associated factors with unhealthy food consumption among students

Longer hours of gaming and television watching were the first common factor for both primary and secondary school students having unhealthy food consumption patterns. A review study conducted by Italian researchers^33^ found a positive association between the time television on and processed meat and junk food consumption tendency. Also, other studies^14^, ^34^ have proven that the more screen time children spend, the higher the chance of unhealthy food intake. School‐aged children who had negative mental health outcomes (depression) were the second common factor for having unhealthy diet habits in our findings. It was in line with a longitudinal study in Taiwan^35^ that adolescents would be emotionally eating when they were depressed by eating more unhealthy foods. Moreover, other studies^36^, ^37^ had gotten the same results as ours that the positive association between depression and unhealthy food consumption. Even though there was a review study^38^ found less significance between these two variables, the relationship between depression and unhealthy food was still critical. Skipping breakfast was the third common factor of unhealthy diet habits for school‐aged children in the present study. It was a common issue among children in other countries,^16^, ^39^, ^40^ as the researchers believed that it was possible due to inadequate sleeping time or getting up late. Nearly 60% of students in the UK and around 25% of students in Greece skipped breakfast when they had insufficient time to rest. Also, the Greek study^16^ had proven that young children who slept less than 8 h per day would increase the chance by 23% (95% CI: 1.20–1.26) of skipping breakfast. The Australian study^40^ suggested a higher prevalence of skipping breakfast when school‐aged children with later bedtimes. Our current finding fills the knowledge gap in the Eastern Asian context, which is also in line with the previous literature in the Western countries.

Usually, a higher prevalence of unhealthy food consumption would align with obesity which was the mainstream in this field of research. However, our study found that young school‐aged students with underweight would eat more unhealthy foods, and it was matched with a Korean study^41^ that underweight children ate more fried food (14.8%) to gain weight. A point needs to be addressed that some young children would eat more unhealthy food to gain weight which may be because of their physical appearance. As they may look tinier than other classmates, or they may need to be stronger for better performance in sports,^42^ or even due to other considerations,^43^ these students would choose to eat more food with high energy or high calories.

Moreover, later bedtime (11 p.m. or later) was not only found with a significant association with elementary school students' eating habits, but also proved by other studies in various countries. Later bedtime among Hong Kong children was found in a cross‐sectional study^44^ with two cities (Hong Kong and Shanghai) comparison. The relationship between later bedtime and unhealthy eating habits was proved by other studies, for instance, a higher likelihood of drinking soft drinks (*β* = −9.2, *p* = 0.01; *β* = 0.23, *p* = 0.001) was found among the young students in the United States,^45^ higher tendency on eating junk food was found among the young students in Australia.^40^

FAS and unhealthy eating among secondary school students, especially male students. Regarding the gender difference in the secondary school setting, female students appeared to consume more fruit and vegetables, limited salt intake, and higher calorie food than male students.^46^ It shared a similar point with our findings that the male gender and poorer mental health were keen to eat more unhealthy food. Besides, a Lithuanian study^47^ found that family socioeconomic status was positively linked with higher intakes of soft drinks and sweets that contain a lot of sugar. This finding supported our analysis that students who were from middle to higher FAS score families would have higher odds of unhealthy food consumption.

### 4.3 Strengths and limitations

This is the first study to comprehensively ascertain the factors associated with unhealthy eating habits in school‐aged children and adolescents in Hong Kong. The bilingual questionnaire spanning academic performance, lifestyle, physical, and psychosocial health was also validated through pilot testing by a wide range of experts. Finally, the large sample size of this study and diverse representation from 65 schools across Hong Kong suggest that factors have been thoroughly and robustly identified. However, in this study, participants were invited to complete the self‐administered questionnaires. Some responses may fail to verify. Although the survey was conducted with the highest confidentiality, due to the given social expectation, the respondents might not provide the true information for this project. Then, as this was a cross‐sectional study, the cause‐effect relationship between the variables might not be certainly determined.

### 4.4 Implications and conclusions

For the first time, this study has quantitatively identified the factors affecting unhealthy eating habits in school‐aged children and adolescents in Hong Kong. Several similarities were found between the factors for unhealthy eating habits demonstrated in Hong Kong as well as other countries. These findings supported the significance of factors that were previously identified, validating these factors in the local context of Hong Kong. Longer hours of leisure time, mental health conditions, and skipping breakfast represent a diverse range of factors that may be addressed at an individual‐ and system level. Further research may design interventions targeting the factors identified from a holistic approach, involving children and adolescents, parents, health professionals, and school staff. Policymakers may also consider designing campaigns and changing policy to more effectively address factors for unhealthy eating behaviors and ultimately lower the proportion of overweight and obese children and adolescents in Hong Kong.

## AUTHOR CONTRIBUTIONS

**Junjie Huang**: Conceptualization; supervision; writing—original draft. **Sze Chai Chan**: Writing—original draft; formal analysis. **Wing Sze Pang**: Writing—original draft. **Shui Hang Chow**: Writing—original draft. **Yat Ching Fung**: Writing—original draft. **Vera M. W. Keung**: Data curation. **Calvin K. M. Cheung**: Data curation. **Amelia S. C. Lo**: Data curation. **Lancelot W. H. Mui**: Writing—review and editing. **Albert Lee**: Writing—review and editing. **Martin C. S. Wong**: Conceptualization; Supervision; writing—review and editing.

## CONFLICT OF INTEREST STATEMENT

The authors declare no conflicts of interest.

## ETHICS STATEMENT

The study was approved by the Clinical Research Ethics Committee (CREC), the Chinese University of Hong Kong (CUHK) and the New Territories East Cluster (NTEC) (CREC‐2013‐632).

## TRANSPARENCY STATEMENT

The lead author Martin C. S. Wong affirms that this manuscript is an honest, accurate, and transparent account of the study being reported; that no important aspects of the study have been omitted; and that any discrepancies from the study as planned (and, if relevant, registered) have been explained.

## Supporting information

Supporting information.

## Data Availability

The datasets used and/or analyzed during the current study are available from the corresponding author on reasonable request.
