# Supplementary material for: Factors associated with consuming unhealthy food in school children: A population‐based study from Hong Kong
Source: Health Sci Rep. 2024 Mar 11;7(3):e1964. doi: 10.1002/hsr2.1964 (PMC10927937; doi:10.1002/hsr2.1964)
Supplement: Supplementary file 1 — Supporting information. [file HSR2-7-e1964-s001.docx]

**Supplementary Legends**

**Supplementary material 1** Questionnaire

**Supplementary material 2** Coding Variables

**Supplementary material 1** Questionnaire

Questionnaire

*1. What is your gender?*

*2. What is your age?*

*3. Do you have your own bedroom? (not including shared room with family members)*

Yes

No

*4. How would you describe your academic performance in the past 12 months? (compared with other students at your grade)*

Excellent

Good

Average

Bad

Poor

*5. What level of expectation do you think your parents have on your academic performance?*

Very high

High

Average

Low

Very low

*6. Does your family own any cars or other vehicles?*

No

Yes, one

Yes, two or above

*7. Have you travelled outside Hong Kong with your family over the past 12 months? (does not include visiting relatives in your hometown or paying tribute to ancestors)*

No

Once

Twice

Three times or more

*8. How many computers do you have at home? (Desktop computers, laptops and tablets)*

Zero

One

Two

Three or more

*9. How would you describe your general health over the past 30 days as*

Very good

Good

Normal

Bad

Very bad

*10. In the past 7 days, how many days did you have physical activity of moderate or vigorous intensity for a total of at least 60 minutes per day?*

0 day

1 day

2 days

3 days

4 days

5 days

6 days

Every day

*11. During the past month, what time have you usually gone to bed at night?*

9:00 pm

9:30 pm

10:00 pm

10:30 pm

11:00 pm

11:30 pm

12:00 am

12:30 am

1:00 am

1:30 am

Other specific time:

*12. Which of the following are you trying to do about your weight?*

I am not trying to do anything about my weight

Stay the same weight

Lose weight

Gain weight

The following questions relate to your exercise habits during the past month only. Your answer should indicate the most accurate reply for the majority of days and nights in the past month.

*13a. In general, how many hours, in total, do you spend on watching TV and online TV and YouTube videos during normal school days?*

I don’t watch TV or video on normal school days

Less than an hour per day

1 hour to less than 2 hours per day

2 hours to less than 4 hours per day

4 hours to less than 6 hours per day

6 hours to less than 8 hours per day

8 hours or more per day

*13b. In general, how many hours, in total, do you spend on playing video games or computer games during normal school days? (Media use can be game consoles, smartphones, tablets or desktop computers etc.)*

I don’t play games or I use computer for my homework on normal school days

Less than an hour per day

1 hour to less than 2 hours per day

2 hours to less than 4 hours per day

4 hours to less than 6 hours per day

6 hours to less than 8 hours per day

8 hours or more per day

*13c. In general, how much time, in total, do you spend on using social networking Apps or social websites during normal school days? For example, Facebook, Instagram, WhatsApp, WeChat, or Blogs*

I don’t use social networking apps or websites on normal school days

Less than an hour per day

1 hour to less than 2 hours per day

2 hours to less than 4 hours per day

4 hours to less than 6 hours per day

6 hours to less than 8 hours per day

8 hours or more per day

The following questions are about your mental health conditions. Your response will never be accessed by anyone other than the researchers.

*14 In the past 12 months, did you ever feel so bad or hopeless almost every day for two weeks or more?*

No

Yes

*15. In the past 30 days, how often did you feel: (None of the time (0), a little of the time (1), some of the time (2), most of the time (3), all of the time (4))*

*15a. Nervous*

*15b. Hopeless*

*15c. Restless or fidgety*

*15d. So depressed that nothing could cheer you up*

*15e. That everything was a burden*

*15f. worthless*

The following questions are about your view of life. Please respond according to your true feelings instead of according to expectations from others or yourself.

Please choose the most appropriate description for your satisfaction with the following aspects:

(Terrible (1), Unhappy (2), Mostly dissatisfied (3), Mixed (Equally satisfied & dissatisfied) (4), Mostly satisfied (5), Pleased (6), Delighted (7))

*16a. Family life*

*16b. school experience*

The following questions are about your dietary habits. Please respond according to your true feelings instead of according to expectations from others or yourself.

During the past 7 days, how often did you eat:

*17.a. Crisps or other snacks (e.g. potato chips, prawn crackers) (~35 grams per packet)*

No

1-3 times in 7 days

4-6 times in 7 days

Once or more a day

*17.b. Chocolate or candies (3 to 5 pieces)*

No

1-3 times in 7 days

4-6 times in 7 days

Once or more a day

*17.c. Desserts, ice-cream, cake or tart (~150 grams per piece or cup)*

No

1-3 times in 7 days

4-6 times in 7 days

Once or more a day

*17.d. Soft drinks (~330 mL per can)*

No

1-3 times in 7 days

4-6 times in 7 days

Once or more a day

*17.e. Carton-packed juice, lemon tea or other sugary drinks (~250mL per carton)*

No

1-3 times in 7 days

4-6 times in 7 days

Once or more a day

*17.f. Fried food (e.g. French fries, fried chicken, etc.)*

No

1-3 times in 7 days

4-6 times in 7 days

Once or more a day

*17.g. Processes or preserved meat (e.g. sausage, ham, BBQ pork, Chinese sausage, etc.)*

No

1-3 times in 7 days

4-6 times in 7 days

Once or more a day

*18. In the past 7 days, on how many days did you eat breakfast?*

Every day

5 to 6 days

3 to 4 days

1 to 2 days

0 days

Not sure

**Supplementary material 2.** Coding Variables

Only variables undergone transformation are shown.

4. How would you describe your academic performance in the past 12 months? (Compared with other students at your grade)

Excellent (0)

Good (0)

Average (1)

Bad (2)

Poor(2)

Description: VAR4 has been transformed into a 3-item categorical data to indicate their self-perceived academic performance: Excellent and good (0), Average (1), bad and poor (2).

7. Have you travelled outside Hong Kong with your family over the past 12 months? (Does not include visiting relatives in your hometown or paying tribute to ancestors)

No (0)

Once (1)

Twice (1)

Three times or more (1)

Description: VAR7 has been transformed into a binary categorical data to indicate having any experience of travelling.

8. How many computers do you have at home? (Desktop computers, laptops and tablets)

Zero (0)

One (1)

Two (1)

Three or more (1)

Description: VAR8 has been transformed into a binary categorical data to indicate the possession of any computer at home

10. In the past 7 days, how many days did you have physical activity of moderate or vigorous intensity for a total of at least 60 minutes per day?

0 day (0)

1 day (0)

2 days (0)

3 days (1)

4 days (1)

5 days (1)

6 days (1)

Every day (1)

Description: VAR10 has been transformed into a categorical data: physically inactive:0-2 days (0), physically active: 5-7 days (1).

11. During the past month, what time have you usually gone to bed at night?

9:00 pm (0)

9:30 pm (0)

10:00 pm (0)

10:30 pm (0)

11:00 pm (1)

11:30 pm (1)

12:00 am (1)

12:30 am (1)

1:00 am (1)

1:30 am (1)

Other specific time:

VAR11: VAR11 has been transformed into a binary categorical data to indicate the bed time before 11pm (0), and at 11pm or later (1)

15. In the past 30 days, how often did you feel: (None of the time (0), a little of the time (1), some of the time (2), most of the time (3), all of the time (4))

*15a. Nervous*

*15b. Hopeless*

*15c. Restless or fidgety*

*15d. So depressed that nothing could cheer you up*

*15e. That everything was a burden*

*15f. worthless*

VAR15: Psychological distress: Presence (13-24), Absence (0-12)

Description: VAR15 based on the K6 scale,^1^ the total score is the summation of the scores of the 6 items. The total score is 24, and the cut-off is 13.^2^

VAR16. Please choose the most appropriate description for your satisfaction with the following aspects:

(Terrible (1), Unhappy (2), Mostly dissatisfied (3), Mixed (Equally satisfied & dissatisfied) (4), Mostly satisfied (5), Pleased (6), Delighted (7))

*16a. Family life*

*16c. school experience*

Description: VAR16a-16b have been transformed into a binary variable: not satisfied:1-3 (0), satisfied:4-7 (1).

18. In the past 7 days, on how many days did you eat breakfast?

Every day

5 to 6 days

3 to 4 days

1 to 2 days

0 days

Description: VAR 18 have been transformed into a binary variable: 0 days – 5 to 6 days (0), Every day: (1).

1. Kessler RC, Andrews G, Colpe LJ, et al. Short screening scales to monitor population prevalences and trends in non-specific psychological distress. *Psychol Med* 2002; **32**(6): 959-76.

2. Kim G, DeCoster J, Bryant AN, Ford KL. Measurement Equivalence of the K6 Scale: The Effects of Race/Ethnicity and Language. *Assessment* 2016; **23**(6): 758-68.
